# Evaluation of the Splash Time Test as a Bedside Test for Hiatal Hernia

**DOI:** 10.14740/gr629w

**Published:** 2014-12-27

**Authors:** Thomas Akesson Lindow, Thomas Franzen

**Affiliations:** a Department of Clinical Physiology, Blekingesjukhuset Karlskrona, Karlskrona, Sweden; b Department of Clinical Physiology, Centralsjukhuset Vaxjo, Vaxjo, Sweden; c Department of Surgery and Department of Clinical and Experimental Medicine, Linkoping University, Norrkoping, Sweden

**Keywords:** Esophagus, Esophageal function, Swallowing, Diagnostic test, Splash time test

## Abstract

**Background:**

Hiatal hernias may present with heartburn, acid regurgitation, dysphagia, chest pain, pulmonary symptoms and globus jugularis. Due to the heterogeneous presentation, there is a need for a simple diagnostic instrument when hiatal hernia is suspected. Hiatal hernia may impair esophageal bolus transportation. The splash time test is a rough measurement of esophageal bolus transportation, where time is measured from the start of swallowing a liquid bolus to the appearance of a “splashing” sound at xiphoid level. We aimed to test the hypothesis that the splash time test is prolonged in patients with hiatal hernia compared to normal subjects.

**Methods:**

In 30 patients with hiatal hernia, time was measured from swallow to splash using audiosignal recording. Thirty healthy subjects were used as controls.

**Results:**

Median time from swallow to splash was 4.9 seconds in the patient group and 4.4 seconds in the control group. Five patients, but none of the controls, performed swallows with absence of splash. Using only absence of splash as a pathological result, sensitivity was 23% and specificity was 100%.

**Conclusion:**

The splash time test is not a sensitive instrument in diagnosing hiatal hernias. The absence of splash, however, seems to be a specific marker of hiatal hernia. Further research is needed regarding which other conditions besides hiatal hernia may cause absence of splash. The splash time test can be replaced by the even simpler “splash test”.

## Introduction

Hiatal hernia is a common problem causing both patient suffering and diagnostic difficulties [1-3]. Besides the commonly appreciated association between hiatal hernia and gastroesophageal reflux with heartburn and esophagitis, hiatal hernias may cause esophageal dysphagia, chest pain, pulmonary symptoms and globus jugularis [4-8]. Due to the heterogeneous presentation of hiatal hernias, there is a need for a simple bedside instrument when hiatal hernia is considered a possible differential diagnosis, especially in clinical situations where endoscopy is not easily accessible, for example in a primary care setting.

Esophagus is a muscular tube consisting of an external layer of longitudinal muscular fibers and an inner layer of circular muscular fibers. The circular musculature performs sequential peristaltic contractions which propel bolus towards the stomach [3, 9]. The longitudinal muscle is less vigorously studied than the circular muscle, but is of great importance for effective bolus transportation [7, 10, 11]. The gastroesophageal junction is composed by the phrenoesophageal ligaments, the diaphragmatic crus, the lower esophageal sphincter, and the sharp angle of His. Together these structures create a one-way valve which prevents reflux of gastric contents into the esophagus [3, 4, 12]. A dilated diaphragmatic hiatus and/or weakening of the phrenoesophageal ligaments enables the distal part of the esophagus, including the lower esophageal sphincter, to be herniated upwards into the thoracic cavity creating a sliding hiatal hernia (type I hiatal hernia) [3, 4]. The diagnosis of hiatal hernia can be made through radiological examination, endoscopy or manometry [13]. In recent years high-resolution manometry has proved to provide a highly specific diagnosis of hiatal hernia [4, 13, 14].

In hiatal hernias, diminished distal opposition of longitudinal muscle contraction from phrenoesophageal attachments may result in impaired swallowing [7, 15]. Furthermore, bolus transport inside the hernia is driven by hydrostatic pressure difference rather than peristaltic contractions (Lin et al referred in [16]). If impaired bolus transit in the esophagus is an indirect sign of hiatal hernia, how could this be measured easily? At the Esophagus Laboratory at Vrinnevisjukhuset, Norrkoping, the esophageal transit time for liquid bolus has been used as a rough measurement of esophageal function, at the laboratory known as “the splash time test”. In most cases, when a liquid bolus passes into the stomach, a clear splash sound can be heard using a standard stethoscope below the xiphoid process. Hypothetically, impaired bolus transportation in hiatal hernias would cause a prolonged splash time test and the loss of peristaltic contractions inside the hernia, perhaps in combination with a widened hiatus, might cause an absent splash sound.

Boiron et al performed a “sophisticated” splash time test when assessing esophageal transit by audiosignal recording [17, 18]. Esophageal transit time was found to be shorter after fundoplication than before in patients with hiatal hernias, but the difference was small [17]. When swallowing in upright position a small difference was detected between patients with hiatal hernia compared to normal subjects, using the same acoustic technique [18]. Swallowing in supine position, where bolus transport is not facilitated by gravity, delays bolus transport [19]. We hypothesized that performing the test in supine position would provide a greater absolute difference in esophageal transit time, thereby increasing clinical usefulness of the test. No previous studies have been made comparing the esophageal transit time in supine position in normal subjects and patients with hiatal hernia.

The splash time test is a cheap, simple, easily interpreted and readily available test without discomfort for the patient.

### Aim

The aim of the study was to test the hypothesis that the time from swallow to splash (the splash time test) is prolonged in patients with hiatal hernia compared to normal subjects when performed in supine position.

## Materials and Methods

**Figure 1:**
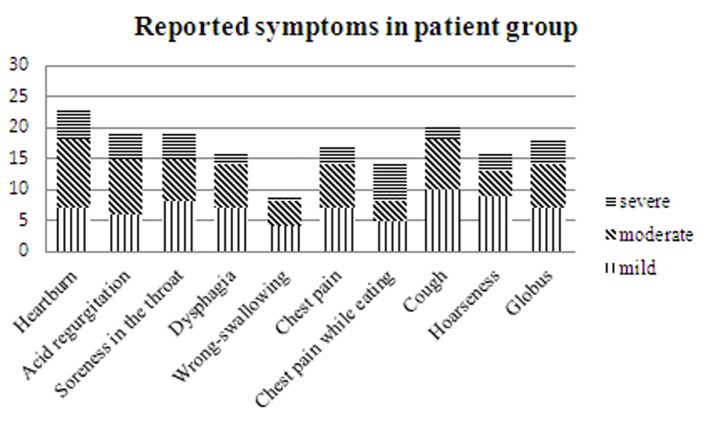
Reported symptoms during the last 2 months, patient group, n = 30.

Thirty patients referred for high-resolution manometry where hiatal hernia was diagnosed, were included in the study. Thirty healthy volunteers without symptoms associated with hiatal hernias were included as controls. The healthy volunteers were recruited among the first author’s friends and co-workers. Baseline characteristics, including sex, age, weight, smoking history and current medications, of all subjects were registered (Table 1) [20]. A questionnaire, regarding presence of heartburn, acid regurgitation, soreness in the throat, globus pharyngeus, chest pain, cough, wrong-swallowing and hoarseness during the last 2 months, i.e. symptoms associated with hiatal hernias, was answered by all participants (Fig. 1). Symptoms were graded from 0 to 3 (none, mild, moderate, severe). Control subjects with positive symptoms were excluded from the study. At the high-resolution manometry study, hiatal hernia was defined as an at least 2 cm separation of the lower esophageal sphincter and the diaphragmatic crus [13].

**Table 1:** Baseline Characteristics

	Patients (n = 30)	Controls (n = 30)
Age (years)	47.4 ± 15.8	47.5 ± 13.6
Length (m)	1.74 ± 9.0	1.74 ± 9.1
Weight (kg)	78.9 ± 16.1	73.7 ± 14.8
Smoker	2	2
Medication LES^†^	3	0
Medication peristalsis^‡^	4	1
Hernia size^§^	3.2 ± 0.75	-
Total reflux time	5.6 ± 9.4	-

Age, length, weight, hernia size and total reflux time are presented as means ± standard deviation. ^†^Medication LES refers to the number of patients on medications that may lower LES pressure (nitroglycerines, beta-receptor agonists, anti-cholinergic drugs, benzodiazepines). ^‡^Medication peristalsis refers to the number of patients on medications that may affect esophageal peristalsis (calcium-channel blockers, nitrates, anti-cholinergic drugs) [20]. ^§^One patient had a fully intra-thoracic ventricle.

All subjects performed three consecutive swallows of 10 mL water boluses in supine position. In order to reduce impairment of esophageal transit due to short swallow intervals, each swallow was separated 30 s [21]. Time was measured from the start of swallowing to the onset of a splash sound at xiphoid level.

In order to increase accuracy in the measurements, audiosignal recording was used. Acoustic data were acquired by two microphones on two levels, cricoid and xiphoid levels. The cricoid microphone was placed on the neck just below the cricoid cartilage. The xiphoid microphone was inserted in a standard stethoscope and the flat diaphragm was placed on the xiphoid cartilage. Cricoid sound represents onset of swallowing and xiphoid sound represents lower esophageal sphincter relaxation (i.e. bolus entry into the ventricle) [18]. Recordings were analyzed using Pro Tools LE (Avid Technology Ltd, Dublin, Ireland) and filtered with a high-pass filter (500 Hz) and a low-pass filter (1.2 kHz). All tests were performed with the subjects on an empty stomach (fasting at least 4 h), and in the patient group prior to the high-resolution manometry study.

### Ethical considerations

Informed consent was gathered from all study participants. Ethical approval was given by the Regional Ethical Review Board in Linkoping (Dnr 2013/82-31).

### Statistical analysis

All statistical analysis was performed with commercially available statistical software, SPSS version 20, IBM, Chicago, IL, USA. Baseline characteristics are described using means and standard deviations. Due to lack of normality in data, splash time test data are described as medians (seconds) and quartiles. Wilcoxon’s rank sum test was used to compare median transit times between the different groups. ROC-analysis was performed to evaluate test accuracy. Spearman correlation test was used to assess correlation between the splash time test and the reported degree of swallowing difficulties. A P-value of < 0.05 was considered statistically significant.

## Results

**Figure 2:**
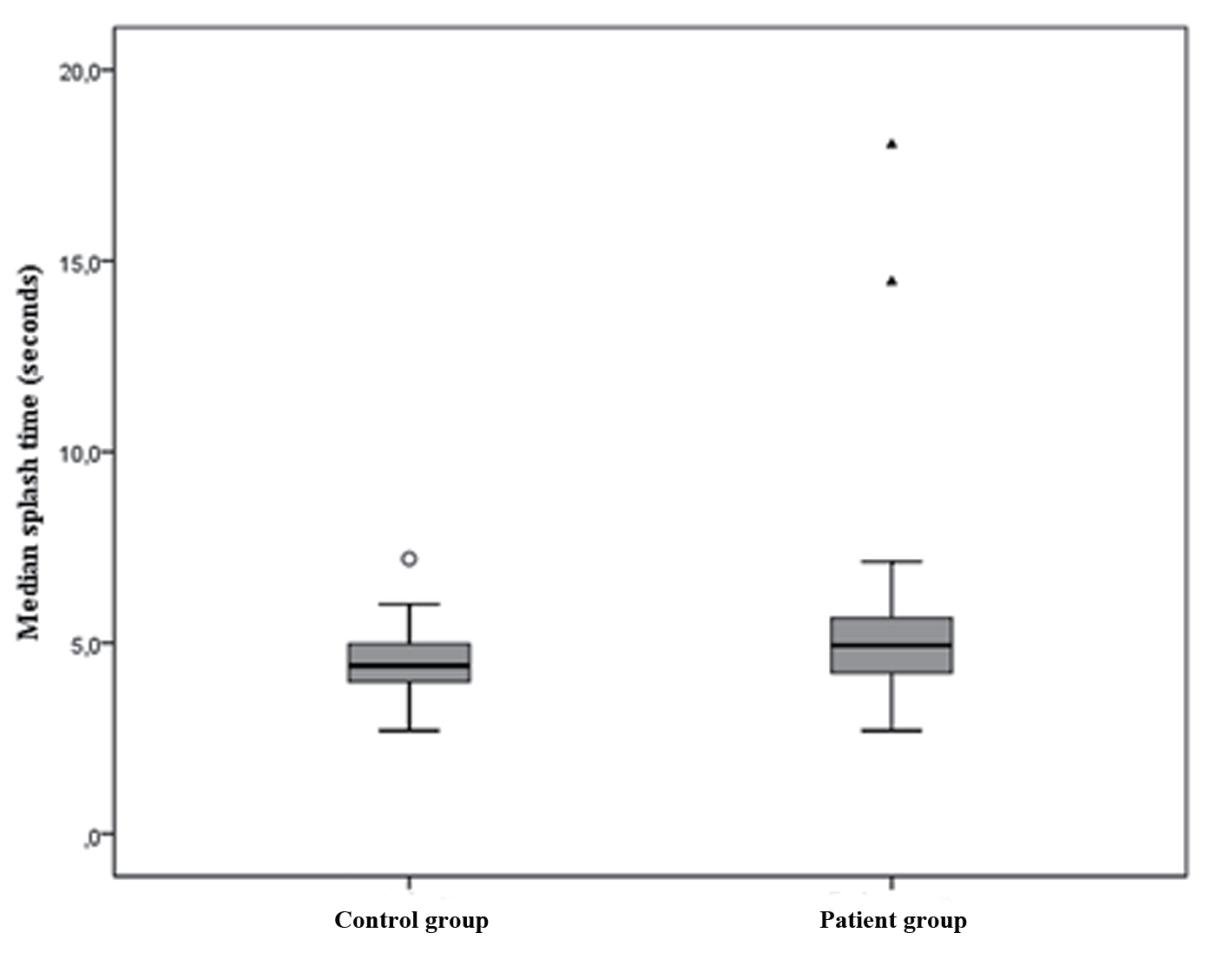
Box-plot presenting median splash time test results in controls (left) and patients (right).

When swallowing a 10 mL bolus, all controls performed successful swallows with good recording quality. In two patients, a splash sound was not heard in any of the three consecutive swallows. Five patients performed swallows where a splash sound was not heard in at least one of the three swallows. The median times from onset of swallow to splash were 4.9 s in the patient group (interquartile range 4.2 - 5.6, min 2.7, max 18) and 4.4 s in the control group (interquartile range 4.0 - 5.0, min 2.7, max 7.2) (P = 0.049) (Fig. 2). The two patients without audible splash sound in any of the recordings were not included in the presentation of medians.

ROC-analysis of splash time test results was performed and absence of splash in all three swallows was included in the analysis as a pathological result. The ROC-analysis yielded an area under the curve (AUC) of 0.68 (0.55 - 0.82). Setting the reference limit of the test to 6 s (maximum in control group, outlier excluded), sensitivity was 27% and specificity was 95%. Using only absence of splash in any of the three swallows as a pathological result, disregarding from the measured splash time, sensitivity was 23% and specificity was 100%.

There was no significant correlation between the splash time test and the reported degree of dysphagia (correlation coefficient 0.2, P = 0.31).

## Discussion

The splash time test is not a sensitive instrument in diagnosing hiatal hernias. However, some of the hiatal hernia patients, but none of the controls, performed a splash time test with absent splash sound. The absence of a splash seems to be a specific marker of hiatal hernia (sensitivity was 23% and specificity was 100%). Further studies regarding the mechanism and the occurrence of absent splash sounds in different esophageal pathologies are needed. The loss of peristaltic contractions inside the hernia perhaps in combination with a widened hiatus might cause an absent splash sound.

The impact of hiatal hernia on swallowing is clearly a complex matter. Even though a small majority of patients in the hiatal hernia group complained of dysphagia, bolus transport, measured with splash time test, was not prolonged in many cases. Also, no correlation was found between the splash time and the reported degree of dysphagia. Dysmotility and prolonged esophageal transit time have been observed in patients with gastroesophageal reflux [16, 22, 23]. Even though the association between hiatal hernia and gastroesophageal reflux disease is well established, discordant results have been presented regarding dysmotility in patients with hiatal hernias [3, 8, 16, 22, 23]. Esophageal bolus transport may be impaired even without manometrically described abnormalities [8, 23].

One of the major weaknesses of this study is that the controls did not perform high-resolution manometry. The risk of including controls with hiatal hernias, though asymptomatic, cannot be disregarded. Estimations of prevalence of hiatal hernia are difficult to interpret. Diagnostic criteria are varying and data generally relate to patients referred for examination, such as endoscopy, rather than asymptomatic subjects. When performing Barium swallow examination in asymptomatic individuals, Dyer and Pridie found hiatal hernias in 33% [24]. The radiographic diagnosis is, however, complicated by difficulties in differentiating the physiologic herniation during swallow and a pathological herniation [3, 13]. In a Swedish population, 14.5% undergoing upper endoscopy were found to have a hiatus hernia [20]. When intra-operative diagnosis of hiatal hernia was used as the golden standard, high rates of false positive results were seen with pre-operative endoscopy (31.7%) [14]. Hiatal hernias may be present in some of the control subjects, but the prevalence is most likely greatly lower than in the patient group making the comparison relevant still, if performed carefully.

In this study, we detected a small but statistically significant difference in medians between groups. Similar results were seen in the work of Boiron et al, where esophageal transit time, assessed by audiosignal recording, was found to be shorter after fundoplication in patients with hiatal hernias, but the difference was small. In two of the 21 patients included, no xiphoid sound was heard [17]. Using the same acoustic technique, esophageal transit times in healthy subjects were slightly shorter compared to patients with hiatal hernias (5.6 vs. 7.2 s) [18]. In these studies, swallowing was performed in upright position. Swallowing in supine position, as performed in our study, did not seem to increase clinical usefulness of the test.

Because of the small difference in median splash time between patients and controls, the splash time test is not a useful instrument in diagnosing hiatal hernias. The even simpler “splash test”, using only water and a stethoscope and “listening” merely after absence of splash, is a cheap, extremely simple, easily interpreted and readily available test without discomfort for the patient. In a patient, for example, with chronic cough where no pulmonary cause have been established or a patient with chest pain without cardiac origin, the simple splash test could be performed bedside. An absent splash would then be able to direct further investigations regarding the presence of hiatal hernia and/or gastroesophageal reflux disease.

In conclusion, the splash time test is not a sensitive diagnostic test for hiatal hernias. The absence of splash, however, seems to be a specific marker of hiatal hernia. The splash time test may therefore be replaced by the even simpler “splash test” but further research is needed to evaluate splash test in asymptomatic subjects where a hiatal hernia is actively excluded. Future studies providing information on which conditions besides hiatal hernia can cause absence of splash and the applicability of the test in clinical situations are also needed.
